# Usnic Acid Potassium Salt: An Alternative for the Control of *Biomphalaria glabrata* (Say, 1818)

**DOI:** 10.1371/journal.pone.0111102

**Published:** 2014-11-06

**Authors:** Mônica C. B. Martins, Monique C. Silva, Luanna R. S. Silva, Vera L. M. Lima, Eugênia C. Pereira, Emerson P. S. Falcão, Ana M. M. A. Melo, Nicácio Henrique da Silva

**Affiliations:** 1 Departamento de Bioquímica, Universidade Federal de Pernambuco, Pernambuco, Brazil; 2 Departamento de Radiobiologia, Universidade Federal de Pernambuco, Pernambuco, Brazil; 3 Departamento de Ciências Geográficas, Universidade de Pernambuco, Pernambuco, Brazil; 4 Centro Acadêmico de Vitória de Santo Antão, Universidade Federal de Pernambuco, Pernambuco, Brazil; Gettysburg College, United States of America

## Abstract

In Brazil, the snail *Biomphalaria glabrata* is the most important vector of schistosomiasis due to its wide geographical distribution, high infection rate and efficient disease transmission. Among the methods of schistosomiasis control, the World Health Organization recommends the use of synthetic molluscicides, such as niclosamide. However, different substances of natural origin have been tested as alternatives for the control or eradication of mollusks. The literature describes the antitumor, antimicrobial and antiviral properties of usnic acid as well as other important activities of common interest between medicine and the environment. However, usnic acid has a low degree of water solubility, which can be a limiting factor for its use, especially in aquatic environments, since the organic solvents commonly used to solubilize this substance can have toxic effects on aquatic biota. Thus, the aim of the present study was to test the potassium salt of usnic acid (potassium usnate) with regard to molluscicidal activity and toxicity to brine shrimp (*Artemia salina*). To obtain potassium usnate, usnic acid was extracted with diethyl ether isolated and purified from the lichen *Cladonia substellata*. Biological assays were performed with embryos and adult snails of *B. glabrata* exposed for 24 h to the usnate solution solubilized in dechlorinated water at 2.5; 5 and 10 µg/ml for embryos, 0.5; 0.9; 1;5 and 10 µg/ml for mollusks and 0.5; 1; 5; 10 µg/ml for *A. salina*. The lowest lethal concentration for the embryos and adult snails was 10 and 1 µg/ml, respectively. No toxicity to *A. salina* was found. The results show that modified usnic acid has increased solubility (100%) without losing its biological activity and may be a viable alternative for the control of *B. glabrata*.

## Introduction

Schistosomiasis is a parasitic disease distributed in 74 countries on the African, Asian and American continents. In Latin America, this disease is found in Venezuela, the Caribbean islands and Brazil. In the latter country, public health agencies reported 0.28 deaths per 100 million inhabitants in 2009, which corresponds to 1.1% of deaths related to infectious diseases [1]. While these data from 2009 seem to indicate a reduction in the transmission of this disease in Brazil, there were 820 cases of the most severe forms between 2000 and 2010, with approximately 505 deaths in the period [2]. It is therefore clear that access of infected individuals to diagnosis and treatment remains limited [3].

Disease transmission is complex due to the many factors involved, e.g., the presence definitive host in the environment, low socio economic status of the population, deficient or absent environmental or household basic sanitation systems, low level of health education among the population at risk and the presence of intermediate hosts (snails) infected with *Schistosoma mansoni* [1]. According to the World Health Organization, control methods for this parasitic disease should involve chemotherapy, health education, basic sanitary services, the interruption of the lifecycle of the parasite and the elimination or reduction in the mollusk intermediate host population in endemic areas using molluscicidal agents [4], [5].

It is estimated that more than 7000 chemicals have been tested for mollusk control, such as copper sulfate, gramoxone, calcium hydroxide, N-tritylmorpholin (Frescon) and niclosamide [6], [7]. Niclosamide is highly efficient in all stages of the mollusk lifecycle, killing 100% of adults and embryos at a concentration of less than 1.5 µg/ml after 2 h of exposure [8]. However, its high cost, sensitivity to sunlight and high degree of toxicity to fish, amphibians and aquatic plants [9] has limited its use, especially in developing countries, which often have serious basic sanitation problems [10]. The use of niclosamide in *B. glabrata* eradications programs is common in Brazil. However, the re-colonization of mollusks is observed after months of application, possibly due to resistance mechanisms developed by these organisms. This makes biological control programs expensive and operationally difficult, especially in poor municipalities [11]. Thus, there is a need for studies aimed at developing specific, low cost alternatives to combating snails. Accordingly, the search for molluscicides derived from plants or natural extracts has gained prominence worldwide, the aim of which is to obtain cheaper, biodegradable, safe, efficient products for controlling snail populations [12].

**Figure 1:**
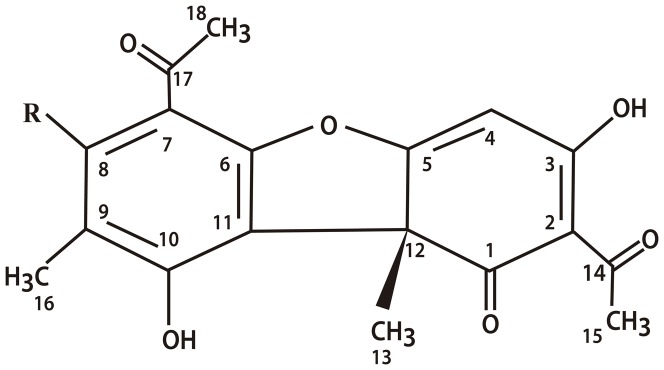
Chemical structure of usnic acid. R =  OH usnic acid; R =  OK potassium usnate.

A lichen is the association of a heterotrophic organism (mycobiont) and an autotrophic organism (photobiont). Lichens produce a variety of substances with phenolic traits, known as lichen substances, which are considered natural protectors of the thallus. The antibacterial [13], antifungal [14], antiviral [15], antitumor [16], insecticidal [17] and sunscreen [18] properties of these substances are known. Phenols derived from lichen substances (some unique to the class) comprise four distinct, well characterized, core chemical structures: depsides, depsidones, depsonesand dibenzofurans (usnic acid) [19]. Usnic acid (Figure 1) is widely distributed among the genera *Cladonia* and *Usnea*, accounting for as much as 6% of the dry weight of the lichen thallus [20]. However, usnic acid has a low degree of water solubility, which can be a limiting factor for its use, especially in aquatic environments, since the organic solvents commonly used to solubilize this substance can have toxic effects on aquatic biota. Potassium and sodium salts derived from usnic acid are completely soluble in water and can be obtained from the purified molecule extracted from different lichens, such as *Cladonia substellata*, or chemically synthesized in the laboratory [21]. The literature describes the antibacterial and fungal properties of these salts [22].

Although usnic acid has been investigated for its potential in different fields of research, including its insecticidal or antiherbivory action on snails, the molluscicidal action of its potassium salt against vectors of parasitic diseases has not yet been described. Thus, the aim of the present study was to investigate the effect of potassium usnate chemically derived from usnic acid purified from *Cladonia substellata* (Vainio) on adults and embryos (blastocyst stage) of *B. glabrata* as well as its toxicity to brine shrimp (*Artemia salina* Leach). The simple bench top bioassay involving the brine shrimp lethality test was used as an environmental bioindicator to probe the pharmacological activity of potassium usnate.

## Material and Methods

### Lichen

Samples of *Cladonia substellata* (Vainio) (300 g) were collected in Mamanguape, Paraíba (northeastern Brazil), at the margins of a Federal Highway BR-101, Coordinates 6° 8′ 19″ S/35° 8′ 11″ W, in August 2012. This way, permission for lichen collection was not required, as well as field studies did not involve endangered or protected species. Thallus identification was performed by MSc Maria de Lourdes Buril and a voucher specimen was deposited at the UFP Herbarium, Dept. of Botany of the Federal University of Pernambuco, Brazil (voucher n° 34402).

### Ether extract to obtain purified usnic acid

The thallus of *C. substellata in natura* (200 g) was maceratedand submitted to four successive extractions with diethyl ether in a Soxhlet apparatus at 40°C for 16 h, as described elsewhere [23].

### Isolation and purification of usnic acid and acquisition of potassium usnate

For the separation, isolation and purification of usnic acid, the ether extract was fractionated in a column of silica gel (70–230 mesh) eluted in the chloroform:hexane (80∶20 v/v) solvent system [24]. After the purification process, 500 mg of the acid were partially dissolved in 40 ml of water at 40°C and subsequently sprayed with previously prepared KOH 10% until complete solubilization of the compound. The solution was frozen at −80°C and then lyophilized.

### Identification of usnic acid and potassium usnate

Usnic acid purity (>95%) was determined using high performance liquid chromatography (HPLC). The HPLC assays involved the use of a Hitachi chromatograph coupled to a UV detector at 254 nm and a reverse phase column (RP 18). The samples (usnic acids purified and usnic standard Merck) were injected at a concentration of 0.1 mg ml. The mobile phase was methanol/water/acetic acid (80:19.5:0.5 v/v/v). The flow was 1.0 ml min^−1^. The substances were identified based on their retention times and comparisons with standard usnic acid [25]. The confirmation of the molecular structure was performed by analyzing the spectra of proton nuclear magnetic resonance (^1^H NMR) obtained at 300 MHz in CDCl_3_ (Varian UNITY spectrometer) and infrared spectroscopy. Potassium usnate was analyzed by infrared spectroscopy (Bruker- IF566).

### *Biomphalaria glabrata* (Say, 1818)

A pigmented wild type strain of *B. glabrata* obtained from São Lourenço da Mata (state of Pernambuco, Brazil) reared in the Laboratory of Radiobiology at the Biophysics and Radiobiology Department at the Federal University of Pernambuco, was used. Adult animals, *S. mansoni* negative, were maintained in plastic aquaria with filtered, dechlorinated water (changed once a week), pH 7.0, at room temperature 25±2°C and fed with fresh lettuce (*Lactuca sativa*).

### Toxicity of potassium usnate to *B. glabrata* embryos

Embryotoxicity assay were conducted with five intact egg masses containing 100 embryos (n = 100) in the blastula stage (15 h after spawning) were selected in a stereomicroscope (Leica MZ6; Leica Microsystems, Wetzlar, Germany) and placed in Petri dishes (10 mm). The potassium usnate (2.5; 5 and 10 µg/ml) or filtered water as control were added to the Petri dishes (10 ml) and, after incubation (24 h). The egg masses were washed under filtrated water and maintained in Petri dishes containing filtered and dechlorinated water at 25±2°C in BOD (Eletrolab). Embryos were observed for 8 days from the beginning of the development until nearly hatching. The mortality and hatched were determined by observation under a stereomicroscope (Leica MZ6; Leica Microsystems, Wetzlar, Germany). The experiments were performed in triplicate for each potassium usnate concentrations.

### Toxicity of potassium usnate to adults of *Biomphalaria glabrata*

Bioassays were performed following the method described by the WHO Expert Committee on the Bilharzia [26]. A population of 200 snails was pre-selected and kept in reproductive isolation for five days to confirm sexual maturity. For the experiments, healthy adult snails (n = 5) of uniform size (shell diameter  = 10±1 mm) were exposed for 24 h to potassium usnate solubilized in filtered water at concentrations of 0.25; 0.5; 0.9; 1; 2.5; 5; 10; 50 and 100 µg/ml with a final volume of 500 ml of solution. The assay was performed in 500 ml glass beakers, and copper (II) carbonate (50 µg/ml) and filtered tap water were used as positive and negative controls, respectively. The snails were considered dead when they showed inactivity, the body and shell were discoloured and absence of heart beats. The bioassays were conducted in triplicate for each of the potassium usnate concentrations.

### Environmental toxicity

Toxicity assay using brine shrimp (encysted eggs of *A. salina* - 25 mg) were hatched in a beaker filled with natural seawater (pH 8), artificial light at 30°C, under constant aeration. After 48 h, the newly hatched larvae were collected and the lethality assay according to procedures described by elsewhere [5]. Groups of 10 (n = 10) larvae were exposed the potassium usnate (0.5; 1, 5; 10 µg/ml) diluted in natural seawater (5 ml) and, after 24 h, the survival rates (%) were recorded. In the control group, larvae were incubated in seawater. The experiments were conducted in quadruplicate for each of the potassium usnate concentrations.

### Statistical analysis

The statistical analysis and standard deviations (SD) were performed using GraphPad Prism 5.0 for Windows (GraphPad Software San Diego, CA, USA) and the data were expressed as replicate means ± SD. Significant differences were established using one-way analysis of variance (ANOVA) and Tukey's test, and p<0.05 was considered significant for both analysis. The lethal concentrations required for 50% killing (LC_50_) of *A. salina* larvae, embryos or *B. glabrata* snails were calculated using probit analysis with a reliability interval of 95% using the StatPlus1 168 2006 software (Analyst Soft, Canada).

## Results

### Chemical analysis

**Figure 2:**
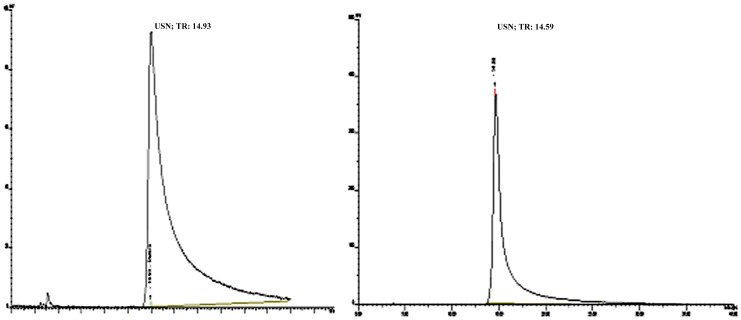
High performance liquid chromatograms. A- Merk usnic acid standard (RT-14.93), B- usnic acid purified from *Cladonia substellata* (RT-14.59).

Purified usnic acid was obtained from the ether extract. The data on the purified substance was analyzed by HPLC (Figure 2), which revealed a retention time (RT) of 14.59 min and 99% purity. The molecular structure confirmed by ^1^H NMR and infrared spectroscopy may be compared with data reported by Huneck and Yoshimura [27], with the following description:^1^H NMR and IR analysis of purified usnic acid: this substances is a yellow crystalline solid, MP, 201–203°C, [α]_D_ at 25°C + 493° (CHCl_3_ c, 1.00), ^1^H NMR (300 MHz, DMSO-d_6_) δH (H; *mult*.; int.): 1.73 (3H; *s*, CH_3_-13), 2.08 (3H; *s*, CH_3_-16), 2.64 (3H; *s*, CH_3_-15), 2.65 (3H; *s*, CH_3_-18), 5.92 (1H; *s*, C-4-H), 11.04 (1H; *s*, C-10-OH), 13.29 (1H; *s*, C-8-OH), 18.89 (1H; *s*, C-3-OH). IR (KBr): 3090, 3007, 2930, 1692, 1632, 1560, 1454, 1370, 1357, 1340, 1330, 1289, 1230, 1190, 1143, 1118, 1070, 1039, 959, 940, 840, 818, 700 cm^−1^.

After modification of the usnic acid to form the potassium salt, the sample was analyzed by infrared spectroscopy (IR) and the following data were obtained: (KBr): 3454, 2982, 2929, 1687, 1631, 1540, 1480, 1460, 1382, 1330, 1291, 1240, 1140, 1188, 1136, 1073, 1038, 980, 940, 880, 840, 820, 770, 730, 700 cm^−1^.

### Toxicity of potassium usnate to *B. glabrata* embryos, adult snails and *A. salina*

**Figure 3:**
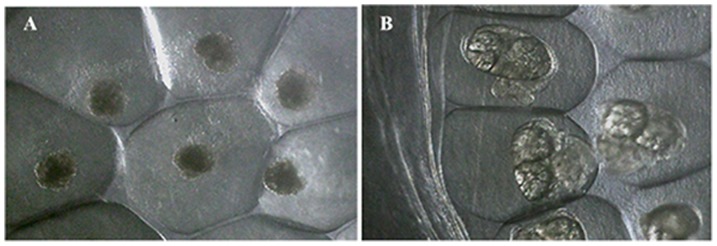
Survival and mortality of *B. glabrata* embryos. A- eggs masses treated at 10 µg ml of potassium usnate showing 100% mortality, B – eggs masses not treated with 100% survival in control group 1 (dechlorinated water).

**Figure 4:**
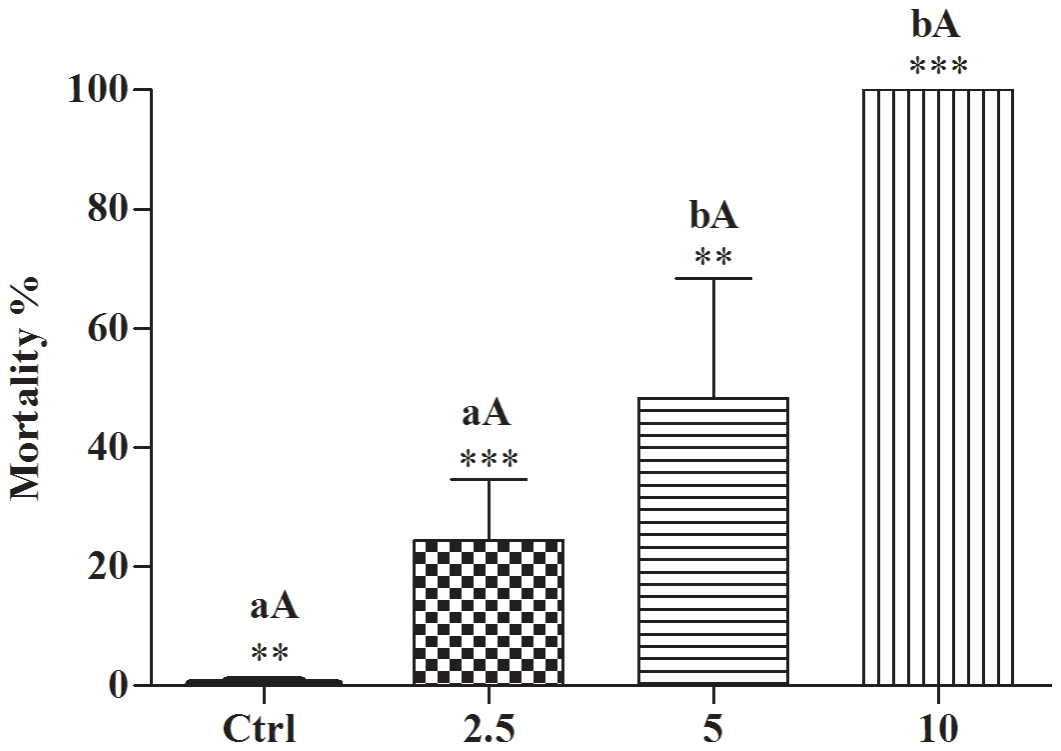
Toxicity of potassium usnate to *B. glabrata* embryos. Mortality of blastula stage embryos subjected to 24 h of treatment with potassium usnate at 2.5; 5 and 10 µg/ml and control group (Crtl 1 – dechlorinated water). Letter A to indicate bars are the same (ANOVA ***p<0.0001); and letter B to indicate that the bars are significantly different from each other (Tukey's test **p<0.05).

The embryos in the blastula stage of development were treated with potassium usnate at 2.5, 5 and 10 µg/ml and the solutions were lethal at all concentrations tested when compared to control groups (p<0.0001, F = 42.46). The mortality rate was 100% at 10 µg/ml (Figure 3). The treatments were significant between the groups: Ctrl vs 5 µg/ml (95% Cl of diff −77,61 to −18.39 **p<0.005), Ctrl vs 10 µg/ml (95% Cl of diff −129.3 to −70.05 ***p<0.0001), 2.5 vs 10 µg/ml (95% Cl of diff −105.3 to −46.05 ***p<0.0001) and 5 vs 10 µg/ml (95% Cl of diff −81.28 to −22.05 **p<0.05) (Figure 4).

**Figure 5:**
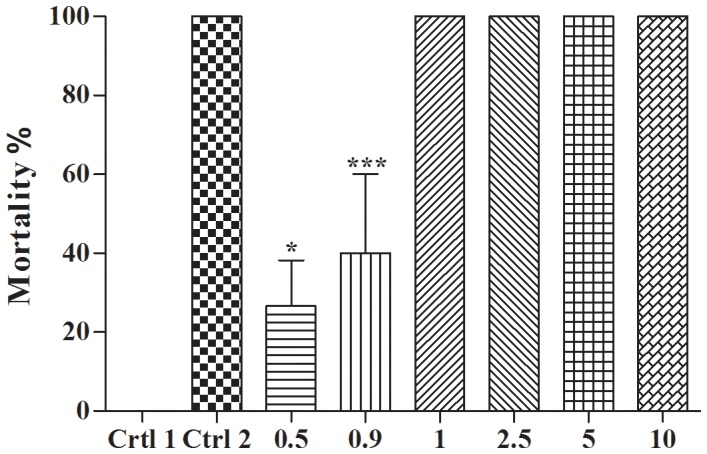
Toxicity of potassium usnate to *B. glabrata* adults. Mortality *B. glabrata* subjected to 24 h of treatment with usnate potassium at 0.5; 0.9; 1; 2.5; 5 and 10 µg/ml and control groups (Crtl 1 dechlorinated water; Crtl 2–cupric carbonate). ***p<0.0001 compared to Ctrl 1 (negative control) group.

Adult snails were more sensitive to the treatments than embryos (***p<0.0001, F = 78.25). At 0.5 µg/ml (95% Cl of diff −49.75 to −3.582 *p<0.05) and 0.9 µg/ml (95% Cl of diff −63.08 to −16.92 ***p<0.0001), mean mortality was less than 100%, but significantly different from the negative control (Ctrl 1) (**p<0.05). The mortality rate was 100% at 1to 10 µg/ml (Figure 5).

**Figure 6:**
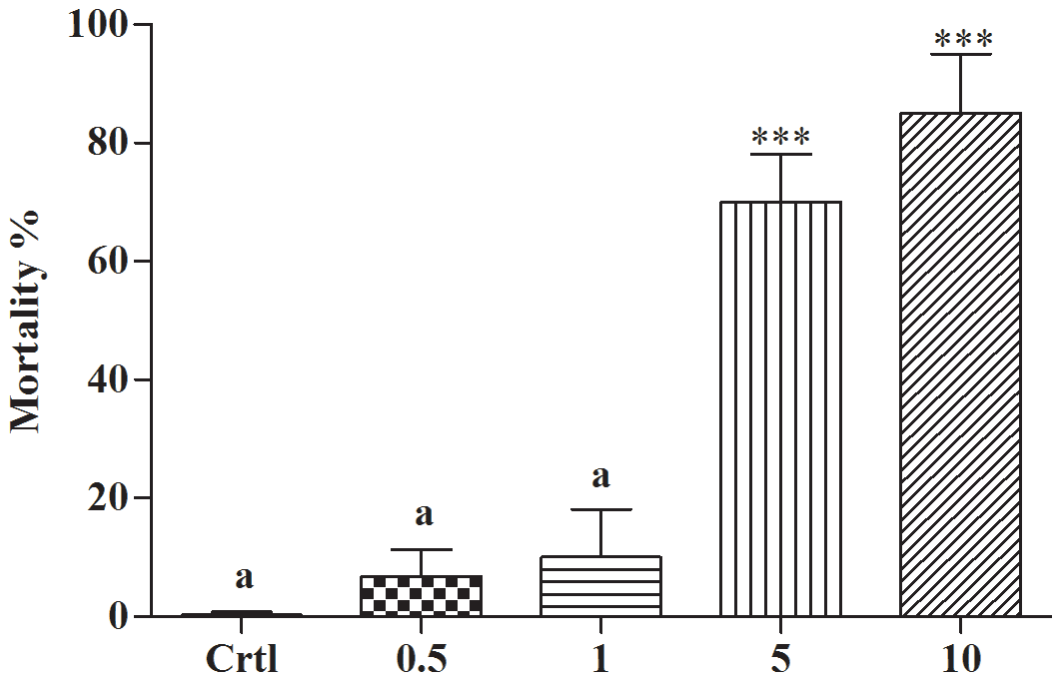
Toxicity of potassium usnate to *A. salina*. Mortality of *A. salina* subjected to 24 h of treatment with potassium usnate at 0.5; 1; 5; 10 µg/ml. ***p<0.0001 for concentrations higher than 5 µg/ml when compared to Ctrl (sea water) group. Letter “a” to indicate that the bars are not significantly different from each other (Tukey's test **p<0.05).

The LC_50_ was 5.77 µg/ml and 0.92 µg/ml for adults and embryos, respectively. In the negative control, no mortality occurred for either embryos or adults, whereas 100% mortality occurred for adult snails treated with cupric carbonate (positive control - Ctrl 2). These results demonstrate the effectiveness of potassium usnate for both embryos and adults of *B. glabrata*. The results of the toxicity on environmental indicator show that potassium usnate was not toxic to *A. salina* at a concentration of 1 µg/ml (95% Cl of diff −25.33 to −5.828), but significant results were found at concentrations above 5 µg/ml(95% Cl of diff −85.33 to −54.17) and 10 µg/ml (95% Cl of diff −100.3 to −69.17) (***p<0.0001) when compared to control group (Figure 6). The LC_50_ for *A. salina* was 2.6 µg/ml.

## Discussion

The mortality rate of *B. glabrata* embryos and adults treated with potassium usnate was higher than that reported for plant extracts. Saponins derived from *Phytolacca icosandra* and *Cartunare gam milotica* are reported to be active at concentrations of 200 to 25 µg/ml and 26 to 3 µg/ml, respectively [28]. The ethanol extract of the latex from *Euphorbia conspicua* (Euphorbiaceae) at a concentration of 4.87 µg/ml is reported to kill 90% of adults within 96 h; the ethanol extract from the leaves of *Solanum jabrense* (Solonaceae) at a concentration of 39.8 µg/ml is reported to kill 90% within at 24 h [29]; and lectins obtained from *Dioclea guianensis* achieve 100% mortality at 10 µg ml^−1^ [5]. However, the whole plant extract of *Agave filifera*, which is rich in flavonoids and steroids [30], is inactive to embryos, likely due to the high molecular weight of their components, which prevents penetration into the gelatinous embryo membrane [31].

A number of studies in the literature report the biological activity of usnic acid isolated from lichens, but few studies have investigated usnate. A line of research has been focused on the antimicrobial activity of sodium usnate against methicillin-resistant *Staphylococcus aureus* [32], whereas no previous reports are found on the molluscicidal activity of potassium usnate. Some studies describe insecticidal and anti-herbivore activities, with records of a deterrent role against herbivores as the most likely ecological function of lichen substances [33], [18]. While lichens do not produce these substances with exclusive deterrent activity against herbivore attacks, some animals, such as the slug *Pallifera varia* (Hubricht.), avoid feeding on lichen species, such as *Xanthoparmelia cumberlandia* (Gyelnik) Hale, which contains usnic, norstictic and stictic acids, and *Huilia albocaerulescens* (Wulfen) Hertel, which produces constictic and stictic acids [34].

Different authors have described the preferences of terrestrial snails that feed on certain thallus parts of lichen and report that invertebrates prefer parts that are free of secondary metabolites [35], [36], [37], [38]. The action mechanisms of lichen substances on snails are not well understood, but the poor palatability and direct toxicity to the intestinal microbiota of invertebrates are likely involved [35], [39]. The studies cited are in agreement with Bustinza [40], who offered pioneering descriptions of the antimicrobial activity of usnic acid. With the observed preference of terrestrial mollusks, it has been hypothesized that the action of the modified molecule in salt form, which is completely water soluble, could have the same toxic effect on aquatic mollusks, as confirmed by the present results.

It is estimated that more than 7000 chemicals have been tested for mollusk control, such as copper sulfate, gramoxone, calcium hydroxide, N-tritylmorpholin (Frescon) and niclosamide [6]. Niclosamide is the agent recommended by the World Health Organization [7] and is highly efficient in all stages of the mollusk lifecycle, killing 100% of adults and embryos at a concentration of less than 1.5 µg/ml after 2 h of exposure [8]. However, its high cost, sensitivity to sunlight and high degree of toxicity to fish, amphibians and aquatic plants [9] has limited its use, especially in developing countries, which often have serious basic sanitation problems [10]. This demonstrates the importance of investigating novel substances of a natural origin that may have the same potential as synthetic products.

The potassium usnate seems to be as effective against mollusk as niclosamide, since 100% of mortality was observed at 1 µg/ml. Therefore, due the high cost and toxic effects of niclosamide on the environment as well as the possible buildup of resistance among snails to this compound have become a constant concern to public health authorities [8]. This underscores the importance of the present findings with regard to combating schistosomiasis.

The *A. salina* test was performed to determine the environmental toxicity of potassium usnate. This microcrustacean is highly sensitive to a number of substances, which favors its use as an experimental model for the evaluation of the toxicity of organic residues that might pose risks to the environment and mammals in general [6]. The assays demonstrated that potassium usnate was non-toxic at the moluscicidal concentration, indicating that usnate offers an extremely low risk to aquatic organisms. These results are in agreement to those reported by Lima et al. [9] on potassium salt (lapachol), which was also found to be non-toxic to brine shrimp.

An extract or active component of a plant molluscicide is only considered effective when achieving 90% mortality at 20 µg/ml for the active ingredient and 100 µg/ml for the extract [4]. The present findings demonstrate that potassium salt from usnic acid has increased water solubility (100%), does not lose its biological activity and may be an alternative drug and prototype for a new class of compounds with moluscicidal activity for the control of *B. glabrata* while exhibiting low toxicity to *A. salina*.

These findings are of considerable importance to countries in which the prevalence of schistosomiasis is high and synthetic chemicals are used to eradicate snails, which are costly and cause serious environmental problems, including the development of other types of diseases in mammals due to their high degree of toxicity. Brazil has a rich lichen flora, particularly in endemic regions for *Schistosoma mansoni*. Thus, further investigation into lichen substances is merited.
